# The race-based stress reduction intervention (RiSE) study on African American women in NYC and Chicago: Design and methods for complex genomic analysis

**DOI:** 10.1371/journal.pone.0295293

**Published:** 2024-04-10

**Authors:** Jacquelyn Y. Taylor, Alexandria Jones-Patten, Laura Prescott, Stephanie Potts-Thompson, Cara Joyce, Bamidele Tayo, Karen Saban

**Affiliations:** 1 Center for Research on People of Color, Columbia University School of Nursing, New York, New York, United States of America; 2 Parkinson School of Health Sciences and Public Health, Loyola University Chicago, Maywood, Illinois, United States of America; 3 Marcella Niehoff School of Nursing, Center for Translational Research and Education, Loyola University Chicago, Maywood, Illinois, United States of America; Dow University of Health Sciences, PAKISTAN

## Abstract

RiSE study aims to evaluate a race-based stress-reduction intervention as an effective strategy to improve coping and decrease stress-related symptoms, inflammatory burden, and modify DNA methylation of stress response-related genes in older AA women. This article will describe genomic analytic methods to be utilized in this longitudinal, randomized clinical trial of older adult AA women in Chicago and NYC that examines the effect of the RiSE intervention on DNAm pre- and post-intervention, and its overall influence on inflammatory burden. Salivary DNAm will be measured at baseline and 6 months following the intervention, using the Oragene-DNA kit. Measures of perceived stress, depressive symptoms, fatigue, sleep, inflammatory burden, and coping strategies will be assessed at 4 time points including at baseline, 4 weeks, 8 weeks, and 6 months. Genomic data analysis will include the use of pre-processed and quality-controlled methylation data expressed as beta (***β***) values. Association analyses will be performed to detect differentially methylated sites on the targeted candidate genes between the intervention and non-intervention groups using the Δ***β*** (changes in methylation) with adjustment for age, health behaviors, early life adversity, hybridization batch, and top principal components of the probes as covariates. To account for multiple testing, we will use FDR adjustment with a corrected p-value of <0.05 regarded as statistically significant. To assess the relationship between inflammatory burden and Δ***β*** among the study samples, we will repeat association analyses with the inclusion of individual inflammation protein measures. ANCOVA will be used because it is more statistically powerful to detect differences.

## Introduction

### Impact of psychosocial stress on DNAm

Changes in DNA methylation (DNAm) may be some of the earliest cellular events in disease onset [1], such aberrant changes have been linked to inflammatory burden [2, 3] and a broad range of diseases including cardiometabolic disease (CMD) [4, 5]. Compelling evidence demonstrates that DNAm is associated with early life adversity [6–8], social stress [9], and history of trauma [10, 11]. Our recent epigenome-wide association study [12] revealed that greater levels of perceived discrimination are associated with decreased DNAm at seven CpG sites linked to inflammatory disease-associated genes in a sample of African American (AA) women [12]. Importantly, DNAm associated with psychological stress is malleable [13], making it a prime target for psychobehavioral interventions [13–16]. To date, only a few studies have explored the impact of psychobehavioral interventions on changes in DNAm of stress response-related genes with the majority focusing on mindfulness interventions. In a small study of veterans with post-traumatic stress disorder who participated in a mindfulness-based stress reduction program, investigators found that, in addition to improvements in trauma symptoms, participants also had an increase in methylation of the FKBP5, a gene involved in modulating glucocorticoid receptor activity [17, 18]. Others have found a decrease in methylation of SLC6A4, a gene related to serotonin transport, following participation in a three-month mindfulness program [19]. In a small randomized, wait-listed control trial, women who participated in an 8-week yoga intervention had reduced methylation of the TNF region as compared to women in the control group [20]. Changes in DNAm have also been associated with tai chi [21], psychotherapy [22], and meditation [23]. The most commonly identified genes associated with DNAm changes following a psychobehavioral intervention include SLC6A4 [19], FKBP5 [22, 24], and BDNF [25, 26].

### Importance of genomics

Genomics has brought significant contributions to cardiovascular disease (CVD) research [27], particularly among AAs, including the combined effects of genetics and cigarette smoking on systolic BP [27] and the effects of body mass index (BMI) on DNAm [28]. DNAm is one type of epigenetic process that modulates gene expression by adding or removing methyl groups to DNA in response to the environment. Studies demonstrate that hyper or hypo methylation of genes due to chronic stressors, including racism and discrimination, are significantly associated with CMD risk [29–36]. Emerging evidence demonstrates that psychobehavioral interventions may modify the methylation of stress response-related genes (e.g., TNF, SLC6A4, FKBP5) [15, 21], potentially buffering the impact of psychological stress at the molecular level. However, few studies have examined the impact of a psychobehavioral intervention on changes in DNAm and none have addressed chronic stress in older AA women.

**Fig 1:**
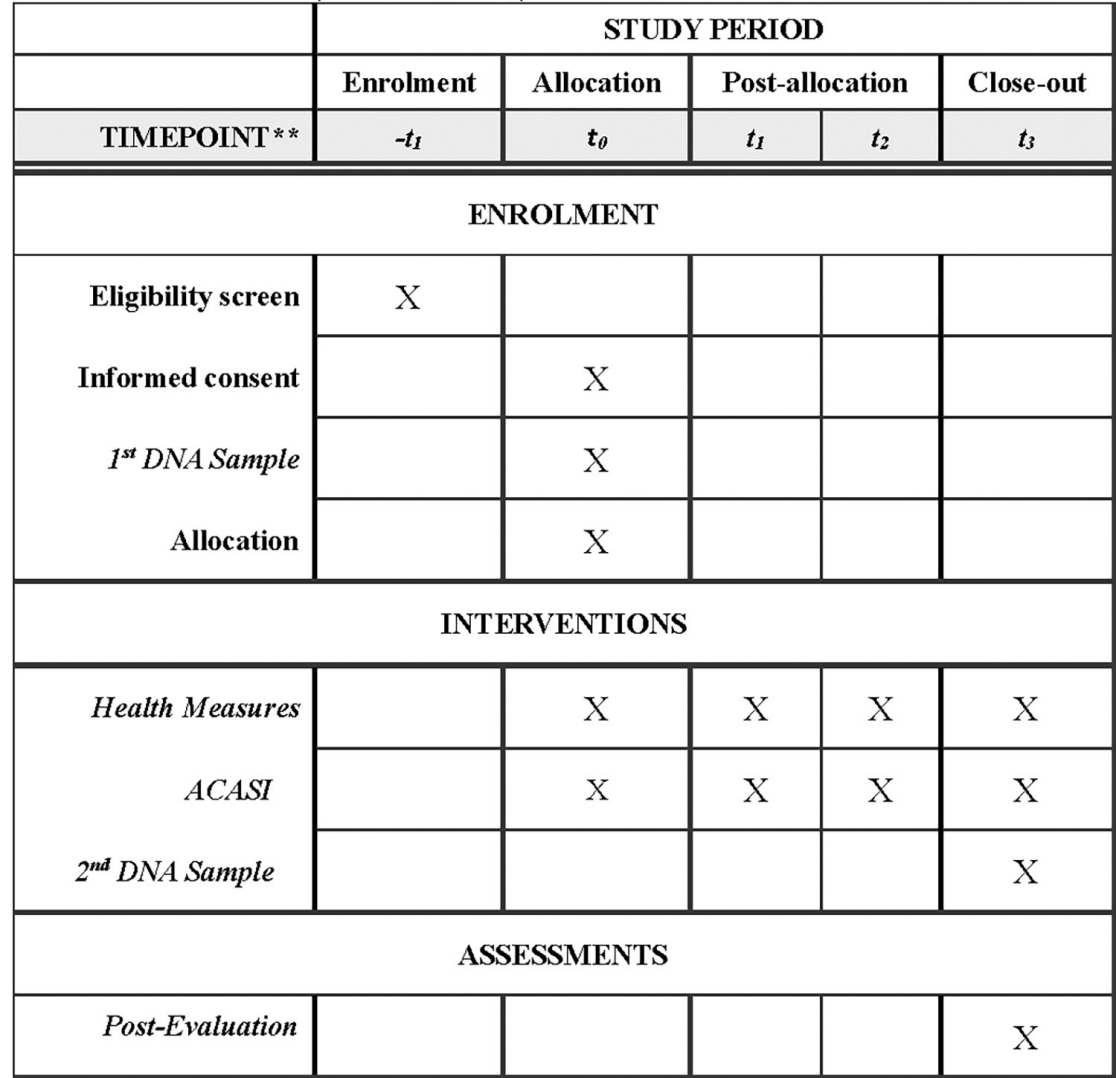
Study period. Schedule of enrollment, interventions, and assessments.

Here we will describe the methods that will be utilized in a randomized clinical trial of AA Women in Chicago and New York City that examines the effect of the RiSE intervention on DNAm pre and post-intervention, and its overall influence on cardiometabolic risk outcomes over time. We will use a targeted candidate gene approach by selecting 20 stress-related candidate genes (and their corresponding CpG sites), assessing DNAm pre and post-RiSE and their association with the outcome measures of cardiometabolic risk (perceived stress, depressive symptoms, and fatigue and sleep disturbance). **See Table 1 and Fig 1.**

**Table 1:** Sample size estimation based on preliminary date. *Assumes n = 125 per group complete cases and *α* = 0.05; r-squared of 5 covariates is 0.30, **The smallest mean difference necessary to ensure > 80% power, assuming pooled SD observed in the pilot.

Outcome evaluated at 6 months	RiSE Mean	Control Mean	|Δ| Means	Pooled SD	Effect size	Power*	Mean difference for 80% power**
	Observed: Pilot Data @ 8 weeks						
WCQ-Active coping	23.76	24.77	1.01	8.24	0.06	21%	2.45
WCQ-Avoidance coping	8.14	14.38	6.24	5.25	0.59	>99%	1.56
WCQ-minimize situation	11.21	14.04	2.83	4.36	0.32	>99%	1.30
DASS-21: Depression	1.45	3.13	1.68	3.09	0.27	>99%	0.92
DASS-21: Anxiety	2.39	3.31	0.92	3.41	0.13	72%	1.02
DASS-21: Stress	4.45	5.90	1.45	4.05	0.18	92%	1.20
TNF-a	0.95	1.00	0.05	0.36	0.07	26%	0.11
High-sensitivity C-reactive protein (hsCRP)	3.83	7.24	3.41	6.30	0.27	>99%	1.88

**Fig 2:**
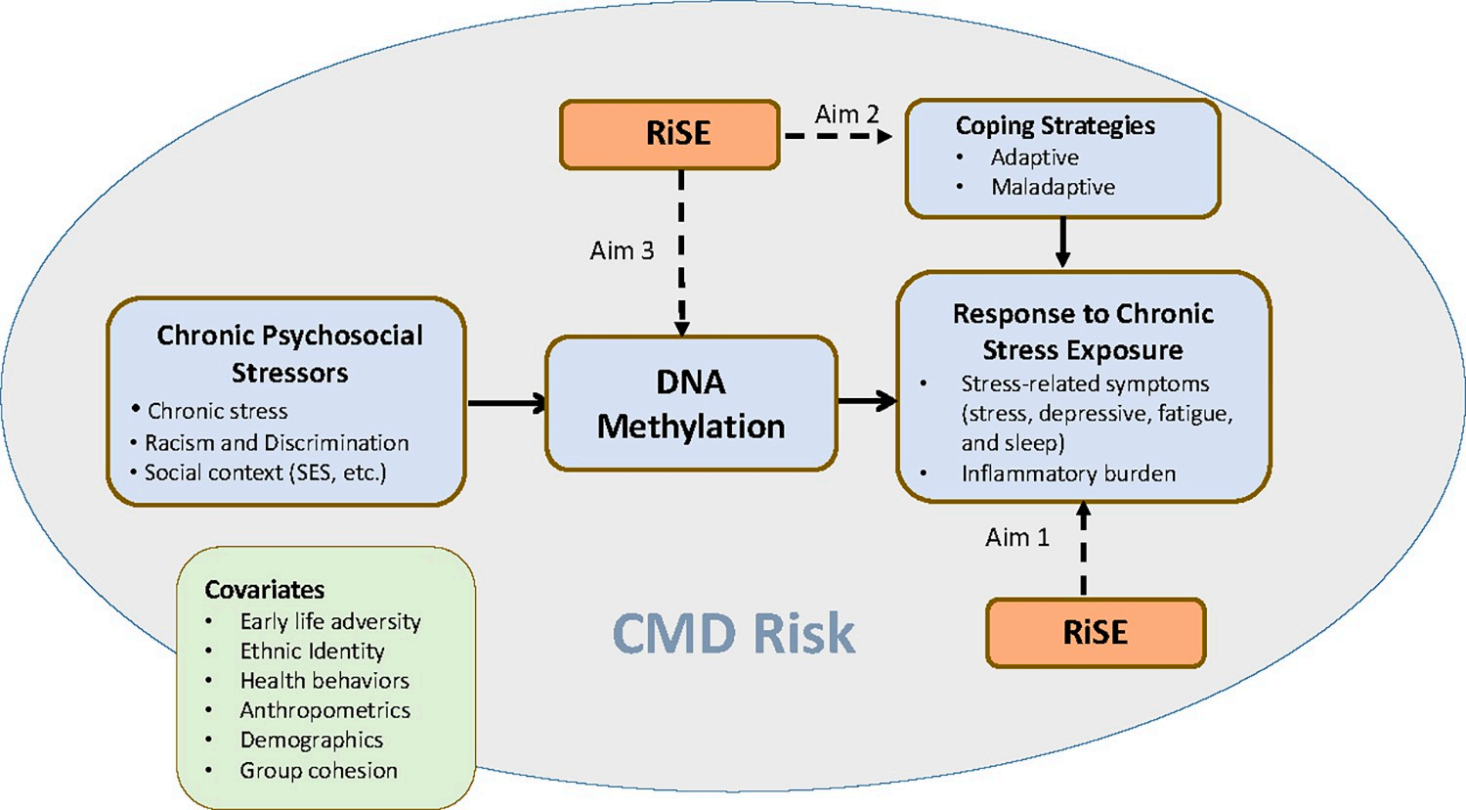
Conceptual model.

*Resilience*, *Stress*, *and Ethnicity* (RiSE) [37–39], is a group-based, 8-week intervention that integrates cognitive-behavioral strategies [40] focused on the biopsychosocial impact of racism [41, 42], racial identity development [43–45], and empowerment [46]. The conceptual model for the study is based on allostatic load theory [47] which posits that accumulating stress or “wear and tear” results in negative physiological consequences. **See Fig 2.**

Based on our preliminary work, we anticipate that participation in RiSE will reduce psychological distress and inflammation. RiSE is a stress reduction intervention that has been shown to be effective in improving coping among AA women [48]. Further, in a targeted candidate gene analysis and epigenomic study in AA women, we found that DNAm patterns of four genes related to the regulation of blood pressure (BP) were associated with stress overload, problem-solving coping, social support coping, and avoidance coping [49], although these associations were not significant after correcting for multiple testing. Others have reported associations between DNAm and stress-related symptoms such as perceived stress, depressive symptoms, fatigue, and sleep [50–52].

Psychosocial stressors, such as discrimination, racism, and social context (e.g., socioeconomic status), when chronic and relentless, can impact the DNAm of stress-response related genes [12, 53], altering the expression of genes that regulate inflammatory response to stress [53]. Based on our preliminary data as well as evidence from other stress reduction interventions [54, 55], we propose that RiSE will improve stress-related symptoms and decrease inflammation (e.g., CRP, IL-6 TNFα, IL-1β, and IFN-γ) [38].

We will use a targeted candidate gene approach by selecting 20 genes (and their corresponding CpG sites) that are associated with our outcome measures to assess DNAm modifications following RiSE (**Table 2**).

**Table 2:** Stress-related candidate genes and functions.

Gene	Function	Gene	Function
FKBP5	Encodes FK506-binding protein 51. Pleiotropic effects on stress and inflammation, associated with CMD [56]	PAC1	Regulates PACAP which is linked to the stress response [57]
SLC6A4	Codes for the serotonin transporter and is associated with depression [58]	5-HTT	Regulation of serotonin transporter protein which is associated with depression and stress [59]
BDNF	Regulates neurogenesis in brain. Alterations of BDNF associated with stress and plays role in regulating body weight [60, 61]	CRF	Regulates homeostasis and neuroendocrine response to stress [62]
TNF	Encodes proinflammatory cytokine. Associated with atherosclerosis [4, 63]	TLR1	Mediates cytokine secretion and inflammatory response to stress [62]
OXTR	Encodes oxytocin receptor and is associated with range of psychological responses [64]	COMT	Instructs enzyme that regulates neurotransmitters associated with stress [65]
IL-6	Regulates proinflammatory cytokine IL-6 [66]	OPRM1	Provides instructions for making mu opioid receptor which is associated with PTSD [67]
NR3C1	Encodes glucocorticoid receptor associated with stress response and CMD [68–70]	KITLG	Inhibitory role on natural killer cells. Associated with cortisol stress reactivity [71]
AVP	Encodes member of vasopressin/oxytocin family. Plays role in glucocorticoid signaling [62]	DNMT-1	Provides instructions for making DNA methyltransferase 1 which is associated with stress response as well as CMD [4, 72, 73]
GABA	Provide instructions for making GABAA receptor protein which produces calming effect [74]	HSD11β2	Implicated in glucocorticoid regulation and response to stress. Associated with CVD [75, 76]
MAOA	Regulates monoamine oxidase A that is related to psychosocial stress [77]	ACE	Regulates angiotensin converting enzyme which is associated with depression and CVD [78]

Our candidate genes were selected based on those consistently associated with stress response in the literature (see **Table 2**). Several studies have demonstrated that DNAm can be modified within weeks to months following changes in environmental factors [79–81]. In those previous studies of longitudinal change of DNAm, a large variation of inter-individual differences over time has been observed [82]. Therefore, our proposed 6-month follow-up not only provides a reasonable time frame to investigate the longitudinal change in DNAm but also links to socio-behavioral stressors among AA women, which have not been previously studied.

### Inflammatory markers

Stress is related to low-grade inflammation, as measured by C-reactive protein (CRP) and proinflammatory cytokines such as interleukin-6 (IL-6), tumor necrosis factor (TNF-α), interleukin-1 beta (IL-1β), and interferon-gamma (IFN-γ) [83–88] as well as increased CMD risk [89]. We will assess the relationship between inflammatory burden IL 6, TNF, IL-1B, IFNG, and CRP and change in methylation among the study samples. See the ‘Genomic Data Analysis’ section below for a description of the analysis with the inclusion of individual inflammatory protein measures.

## Methods

This research was approved by Loyola University’s Institutional Review Board (IRB) Protocol number LU214133 WCG IRB #20230283. This study is registered through ClinicalTrials.gov, registration number: NCT05902741. It is estimated that the recruitment of participants will begin summer of 2023.

### Procedures

Detailed descriptions of this study have been previously discussed [90]. Briefly, this convenience sample will include 300 self-identified AA women residing in either the Chicagoland (n = 150) or New York City (n = 150) area between the ages of 50 and 75 years with risk factors for CMD [91]. Participants will be randomized to either an 8-week RiSE intervention or a Health Education Program (HEP) (attention control). Potential participants will be pre-screened for eligibility over the phone and will be followed by an appointment for further screening if the potential participant is eligible. After collecting informed consent for obtaining inflammatory markers and DNAm samples, the samples will be stored in a lab for processing and frozen for later batch analysis. DNAm samples will be stored at 4°C. Specimens will be shipped overnight on dry ice to respective labs for analysis per best practice guidelines [92]. Individuals will contribute data for up to four-time points (baseline, 4 weeks (mid-intervention), 8 weeks (completion of intervention), and 6 months post-intervention). Data will be collected from participants using an Audio Computer-Assisted Self-Interview system (ACASI) [93]. Perceived stress, depression, fatigue, and sleep disturbance symptoms will be analyzed via the Perceived Stress Scale (PSS-10) [94], Patient Health Questionnaire 9 (PHQ 9) [95], NIH PROMIS Fatigue Short Form 8a [96], and NIH PROMIS Short Form v1.0 [97].

### DNA sample collection

Salivary DNAm will be measured at baseline and 6 months following the intervention. Saliva was selected as the sample of choice as it correlates well with DNAm in blood [98–100], is more similar to patterns of DNAm in the brain as compared to blood [101–102], associates with CMD indices [103], and correlates with frontolimbic brain function [104, 105] and therefore, potentially offering distinct opportunities for studies considering psychological measures function [104, 105].

Participants will collect saliva samples using the Oragene-DNA (OG-510) kit that requires them to spit saliva into a tube up to the 1mL mark. Taylor et al. have described details of DNA collection and analysis procedures elsewhere [106]. Samples will be refrigerated at 4 C° in a lab until DNA extraction and analysis are completed. All analysis of DNA extraction will be done at Columbia University under the guidance of Dr. Taylor. Samples collected at Loyola University will be shipped at ambient temperature to Columbia University for processing and analysis. All tubes and plates that contain an individual’s DNA will be labeled with a barcode to ensure precise sample tracking and recorded in the laboratory’s computerized freezer inventory via barcodes upon arrival at the laboratory.

### Genotyping

The Illumina Infinium Global Diversity Array (GDA) will be used to assess the targeted candidate genes (**Table 2**) and for ancestry informative markers (AIMS). AIMS are included on the GDA chips for multiethnic populations which is suited for our sample of AA women. The GDA combines highly optimized multiethnic, genome-wide content with curated clinical research variants. We selected the GDA chip because it has expanded coverage of SNPs for multiethnic populations [106].

### DNAm analysis

The Illumina Infinium Methylation EPIC v2.0 (900K) BeadChip will be used for examining the methylation of targeted candidate genes [107]. We will apply standard quality control measures including accounting for cell type, batch, and plate effects. This BeadChip directly quantifies DNAm at 936,866 CpG dinucleotides, giving near complete coverage of known genes [107–109]. We will perform hybridization on a per-sample basis. The Infinium arrays are well annotated for CpG dinucleotides in CpG island and non-CpG island promoters, shore regions, coding regions, repetitive elements, miRNA promoter regions, FANTOM5 enhancers, ENCODE open chromatin and enhancers, and DNase hypersensitivity sites and include 91.1% of the loci from the HumanMethylation450 BeadChip. DNAm is determined at each of the CpG sites on the 935K array by measuring the fluorescent signals from the M (methylated) and U (unmethylated) probes specific for each site included in the array, covering approximately 99% of all RefSeq genes and 96% of CpG islands [110]. We will confirm DNAm by methylation-specific polymerase chain reaction (PCR) and by bisulfite sequencing [111]. We may explore the use of additional methylation data in the future to identify additional novel individual genes with abnormal gain and/or loss of CpG methylation associated with social and CMD features for future studies. Additional data from the BeadChip will be retained for future analyses as new information regarding epigenomic differences associated with CMD risk and social factors are described in the literature.

### Data analytic plan

#### Power analysis

**Fig 3:**
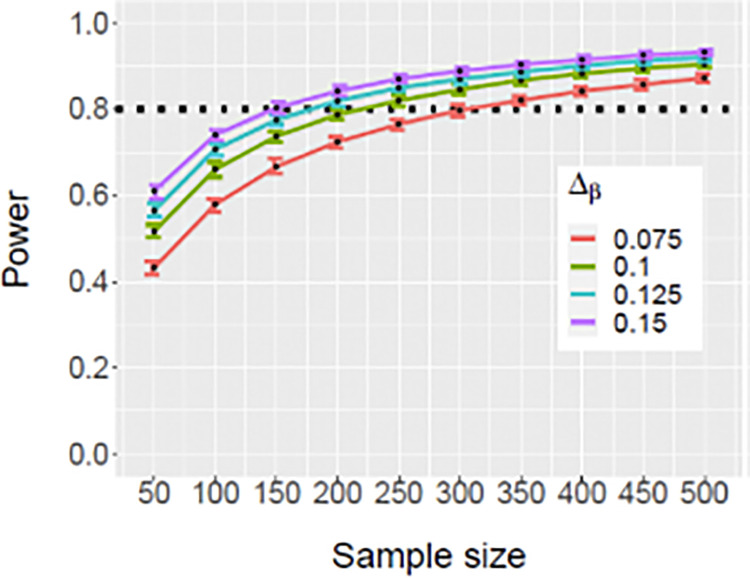
Plot of mean simulated power with 95 percentile (2.5 & 97.5%) for select DNAm differences.

We will focus primarily on the tested CpG sites that are true positives, i.e., detected CpG sites with meaningful differential DNAm. There is currently no standard threshold for meaningful differential DNAm, though previous studies have used thresholds of 5–20% [112] for meaningful differential DNAm, and for this study, we use a conservative threshold of 10%. Using the R package ‘pwrEWAS’ [113], we performed simulations to evaluate sample sizes required to detect effect sizes (percent differences) of 7.5%, 10%, 12.5%, and 15% in CpG-specific methylation across 4000 CpGs on our 20 candidate genes with at least 80% power. We need 200 total subjects (150 per group) to detect a difference of at least 10% with at least 80% power across a set of CpG sites (**Fig 3**). Our simulations also indicated that the power to detect at least one differentially methylated CpG out of the set of 4000 is above 85%.

Data quality and integrity will be ensured before the conduct of statistical analysis. Prior power analyses for each planned analysis were conducted to ensure that each analysis was appropriately powered. All post-hoc tests will be controlled for multiple comparisons (i.e., Type I error). ANCOVA will also be used because it is better at controlling for chance imbalances in randomized groups at baseline, avoids regression to the mean issues, and is generally more statistically powerful in detecting differences [114].

#### Genomic data analysis

Using the pre-processed and quality-controlled methylation data expressed as beta values which are the percentage methylation at each CpG site at specific targeted candidate genes, we will determine changes in methylation (Δ***β***) at every site as the difference between methylation at baseline and methylation at post-intervention. Negative Δ***β*** indicates a decrease in methylation from baseline to post-intervention, and a positive value indicates an increase in methylation from baseline to post-intervention. Association analyses will be performed to detect differentially methylated sites on the targeted candidate genes between the intervention and non-intervention groups using the Δ***β*** with adjustment for age, health behaviors, early life adversity, hybridization batch, and top principal components of the probes as covariates. To adjust for multiple testing, we will use False Discovery Rate (FDR) adjustment with an FDR of <0.05 regarded as statistically significant. All analyses will be performed in R software using the Bioconductor R package ‘limma’ [115]. To assess the relationship between inflammatory burden (IL-6, TNF-α, IL-1β, IFN-γ, CRP) and Δ***β*** among the study samples, we will repeat the above association analyses with the inclusion of individual inflammatory protein measure.

## Discussion

### Potential pitfalls, alternative approaches

Given the longitudinal nature of this study and the significant commitment necessary to participate in the study, attrition is an inherent potential issue. In our preliminary work, we found an 11% attrition rate with a similar design. For this study, we will conservatively over-sample by 20% to accommodate potential attrition to achieve n = 250 participants with complete data at 6 months. We will minimize attrition by collecting multiple phone and email/text messaging contact information for participants and reviewing this information at each visit. It is possible that some participants may not have access to the internet or have technical challenges with Zoom. We will provide iPads and internet access for participants who lack equipment and/or internet access. In addition, our study team will teach participants how to use Zoom as needed and be available in real time to address technical issues. To note, synchronous online platforms have successfully been used to administer group-based psychobehavioral interventions, including in older populations [116–119]. Further, we have experience successfully conducting research groups via Zoom (Saban: PCORI). Despite these limitations, the proposed study will be designed and implemented with the highest degree of scientific rigor and has the potential for significant public health impact. Finally, by providing concrete recommendations based on the results of this intervention, we believe that this is the first study to provide the needed science to implement interventions focused on reducing the impact of multiple forms of stressors on the CMD health of AA women by exploring the biological changes in DNAm and inflammation that may contribute to this disparity.

### Future directions

Lifestyle interventions can take 24 months to impact CMD risk [120] so likely would not be able to capture improvements in CMD risk indices in this proposed study. RiSE may benefit from larger epidemiological samples, other minority populations, and more frequent biological sample collection to detect changes in DNAm and inflammatory burden over longer periods of time. Future studies should evaluate the long-term impact of RiSE on CMD as well as on other inflammatory-related disease outcomes.
